# Effect of Sleep Changes on Health-Related Quality of Life in Healthy Children: A Secondary Analysis of the DREAM Crossover Trial

**DOI:** 10.1001/jamanetworkopen.2023.3005

**Published:** 2023-03-15

**Authors:** Rachael W. Taylor, Jillian J. Haszard, Rosie Jackson, Silke Morrison, Dean W. Beebe, Kim A. Meredith-Jones, Dawn E. Elder, Barbara C. Galland

**Affiliations:** 1 Department of Medicine, University of Otago, Dunedin, New Zealand; 2 Biostatistics Centre, University of Otago, Dunedin, New Zealand; 3 Department of Neuropsychology, Cincinnati Children’s Hospital Medical Centre, Cincinnati, Ohio; 4 Department of Paediatrics and Child Health, University of Otago, Wellington, New Zealand; 5 Department of Women’s and Children’s Health, University of Otago, Dunedin, New Zealand

## Abstract

**Importance:**

Little is known regarding the effect of poor sleep on health-related quality of life (HRQOL) in healthy children.

**Objective:**

To determine the effect of induced mild sleep deprivation on HRQOL in children without major sleep issues.

**Design, Setting, and Participants:**

This prespecified secondary analysis focused on HRQOL, a secondary outcome of the Daily Rest, Eating, and Activity Monitoring (DREAM) randomized crossover trial of children who underwent alternating weeks of sleep restriction and sleep extension and a 1-week washout in between. The DREAM trial intervention was administered at participants’ homes between October 2018 and March 2020. Participants were 100 children aged 8 to 12 years who lived in Dunedin, New Zealand; had no underlying medical conditions; and had parent- or guardian-reported normal sleep (8-11 hours/night). Data were analyzed between July 4 and September 1, 2022.

**Interventions:**

Bedtimes were manipulated to be 1 hour later (sleep restriction) and 1 hour earlier (sleep extension) than usual for 1 week each. Wake times were unchanged.

**Main Outcomes and Measures:**

All outcome measures were assessed during both intervention weeks. Sleep timing and duration were assessed using 7-night actigraphy. Children and parents rated the child’s sleep disturbances (night) and impairment (day) using the 8-item Pediatric Sleep Disturbance and 8-item Sleep-Related Impairment scales of the Patient-Reported Outcomes Measurement Information System questionnaire. Child-reported HRQOL was assessed using the 27-item KIDSCREEN questionnaire with 5 subscale scores and a total score. Both questionnaires assessed the past 7 days at the end of each intervention week. Data were presented as mean differences and 95% CIs between the sleep restriction and extension weeks and were analyzed using intention to treat and an a priori difference in sleep of at least 30 minutes per night.

**Results:**

The final sample comprised 100 children (52 girls [52%]; mean [SD] age, 10.3 [1.4] years). During the sleep restriction week, children went to sleep 64 (95% CI, 58-70) minutes later, and sleep offset (wake time) was 18 (95% CI, 13-24) minutes later, meaning that children received 39 (95% CI, 32-46) minutes less of total sleep per night compared with the sleep extension week in which the total sleep time was 71 (95% CI, 64-78) minutes less in the per-protocol sample analysis. Both parents and children reported significantly less sleep disturbance at night but greater sleep impairment during the day with sleep restriction. Significant standardized reductions in physical well-being (standardized mean difference [SMD], −0.28; 95% CI, −0.49 to −0.08), coping in a school environment (SMD, −0.26; 95% CI, −0.42 to −0.09), and total HRQOL score (SMD, −0.21; 95% CI, −0.34 to −0.08) were reported by children during sleep restriction, with an additional reduction in social and peer support (SMD, −0.24; 95% CI, −0.47 to −0.01) in the per-protocol sample analysis.

**Conclusions and Relevance:**

Results of this secondary analysis of the DREAM trial indicated that even 39 minutes less of sleep per night for 1 week significantly reduced several facets of HRQOL in children. This finding shows that ensuring children receive sufficient good-quality sleep is an important child health issue.

**Trial Registration:**

Australian New Zealand Clinical Trials Registry: ACTRN12618001671257

## Key Points

**Question:**

Does losing sleep affect health-related quality of life in children?

**Findings:**

In this secondary analysis of a randomized crossover trial involving 100 healthy children aged 8 to 12 years, receiving 39 minutes less sleep per night resulted in significantly lower physical and overall well-being, ability to cope well at school, and total health-related quality of life, especially in children with at least a 30-minute difference in sleep.

**Meaning:**

Findings of this secondary analysis of a randomized clinical trial indicate that ensuring children receive sufficient good-quality sleep is an important child health issue.

## Teaser

This secondary analysis examines the self-rated aspects of health-related quality of life, including physical and psychological well-being, social and peer support, and coping at school, among healthy children who were enrolled in a sleep manipulation trial.

## Introduction

While inadequate or poor-quality sleep has been associated with a wide range of adverse physical and psychological health outcomes in infants, children, and adolescents,^1,2,3,4,5^ interest is growing regarding the association of sleep with more global indices of health, such as health-related quality of life (HRQOL).^6^ The HRQOL is a widely used concept with many definitions and measures^7^ and generally encompasses 3 main domains: physical, mental, and social health.^8^

To date, most research has examined the association of more medically related sleep issues, such as obstructive sleep apnea,^9^ insomnia,^10^ and other sleep disorders,^11^ with HRQOL in clinical samples of children or has included outcomes that are only indirectly associated with HRQOL, such as cognitive testing^12^ and mood regulation.^13^ Evidence is emerging that HRQOL is also adversely affected in community samples of children with milder sleep health issues, such as parent-reported sleep problems^14,15^ or sleep initiation or maintenance difficulties.^16^ However, little research has determined whether HRQOL is a factor in sleep in otherwise healthy children. A 2016 systematic review^6^ reported an inverse association between sleep duration and HRQOL in 3 studies but rated such evidence as very low quality. Although several recent large studies have reported associations between sleep and HRQOL in children,^17,18,19^ these studies have all been cross-sectional and unable to determine causality. Few studies have used objective measures of sleep, which can differ markedly from measures obtained from questionnaires.^20,21^ As a recent study showed, device-measured sleep had no association with HRQOL, whereas self-reported ratings of poor sleep quantity and quality were associated with lower HRQOL.^19^

To our knowledge, no experimental studies have yet determined the effect of manipulating sleep on HRQOL in healthy children to the extent that might represent the levels of mild sleep deprivation that many children may experience today.^22^ The aim of this secondary analysis was to determine the effect of mild sleep deprivation (induced via the Daily Rest, Eating, and Activity Monitoring [DREAM], a home-based sleep manipulation trial) on HRQOL in children without major sleep issues.

## Methods

### Study Design

The DREAM randomized crossover trial investigated how mild sleep deprivation influenced eating behaviors and activity patterns in children aged 8 to 12 years in the naturalistic home environment. Detailed information on this trial is provided in the protocol^23^ (Supplement 1) and a previous article on the main outcomes.^24^ This prespecified secondary analysis focused on HRQOL, a secondary outcome of the trial. No sample size calculations were undertaken because HRQOL was a secondary outcome, but this study was sufficiently powered to detect relevant differences in the primary outcomes.^23,24^ The DREAM trial was approved by the University of Otago Human Ethics Committee, and written informed consent (by parents or guardians) or assent (by children) was obtained at the first visit after a verbal explanation of the protocol and an opportunity to ask any questions. We followed the Consolidated Standards of Reporting Trials (CONSORT) reporting guideline.^25^

### Participants

Healthy children were recruited by advertisement between October 2018 and March 2020 and were eligible to participate if they were aged 8 to 12 years; lived in the wider Dunedin area in New Zealand; had no underlying medical conditions or medications that could affect sleep; and scored 39 or lower, which indicated no major sleep problems, on the Sleep Disturbance Scale for Children.^26^ Only children with parent (or guardian)-reported time in bed of 8 to 11 hours per night were included to ensure that the intervention (restriction and extension of time in bed) did not place them in the not recommended category of sleep duration for this age group (ie, <7 or >12 hours per night).^27^ Parents and children were emailed separate written information sheets prior to enrolling.

### Randomization and Masking

Children were randomized to the order in which they underwent sleep restriction and sleep extension weeks and were stratified by age group (8-10 years or 11-12 years) and sex (male or female). Randomization to order was generated by one of us (J.J.H., the study biostatistician) using random block lengths in Stata, version 17.0 (StataCorp LLC) in a 1:1 allocation and then was uploaded to a research management program randomization module (REDCap; Vanderbilt University).^28^ Randomization was undertaken by the research staff following baseline measures. Participants or intervention deliverers could not be blinded to the intervention group (sleep extension or sleep restriction), but those who were analyzing the accelerometry data and the biostatistician were all blinded to the intervention allocation and worked using code A or B to denote group randomization order rather than sleep extension or restriction.

### Procedures

To achieve mild sleep deprivation, children were asked to go to bed 1 hour earlier than usual for 1 week (sleep extension) and 1 hour later than usual for 1 week (sleep restriction), separated by a 1-week washout to allow sufficient time for children to return to their usual sleeping habits before the next intervention week.^29^ Means of usual bed and wake times, determined from a 7-day sleep diary at baseline, were calculated separately for weekdays and weekend days. Researchers discussed with parents whether these means reflected usual bed and wake times and adjusted the means if required (eg, if a child was late to bed on 1 night, this was adjusted to usual as indicated by the parent). Wake times were kept constant to mirror daily life restrictions (eg, school start times), and interventions were administered only during the school term.

Researchers worked with families during a single problem-solving session (typically 5-10 minutes) to identify any barriers to changing bedtimes, such as being at a sporting activity until close to the extension bedtime, that limited the ability to complete prebed activities in a timely fashion. A suggestion to counteract this barrier could have been to prepare the dinner meal earlier that day or to complete school assignments in the morning. Families received daily personalized bedtime text reminders during each intervention week.

### Measures Collected at Baseline

Demographic data were obtained from the parent, including age, sex, and race and ethnicity of the child; presence of any siblings; and maternal educational level (the index used in New Zealand; and all participating parents or guardians identified as mothers). Area-level deprivation for a family was measured with the 2018 New Zealand Index of Deprivation, an index based on the New Zealand Census of Population and Dwellings data that reflects the extent of material and social deprivation used to construct deciles from 1 (least deprived) to 10 (most deprived).^30^ Parents completed the 22-item Children’s Sleep Hygiene Scale,^31^ which assesses the regularity of behaviors that might support or interfere with sleep and has a score range of 1 to 6 for each item, with higher scores indicating better sleep hygiene (subscale and total scores are calculated as means). This information was mainly collected to provide feedback to families but was occasionally used to inform discussions about bedtimes if an individual sleep hygiene practice was uncommon. Duplicate measures of height (in centimeters) and weight (in kilograms) were collected at baseline using standard procedures,^23^ and body mass index *z* scores were calculated using the World Health Organization Child Growth Reference data.^32^

Children wore an accelerometer (ActiGraph wGT3X-BT; ActiGraph LLC), which was set to 30 hz at initialization and downloaded with 15-second epochs, on their right hip 24 hours a day for 1 week to measure sleep timing (onset and offset), sleep duration (total sleep time), and sleep quality (number of awakenings, wake after sleep onset, and sleep efficiency). Actigraphy data were analyzed using a count-scaled algorithm developed in MATLAB (MathWorks Inc) that estimated sleep onset and offset as well as awakenings that were specific to each individual for each day.^33,34^

### Measures Collected During Each Intervention Week

Actigraphy data were collected for each child for the duration of each intervention week, as occurred at baseline. Questionnaire data were collected at the end of each intervention week (day 8) during an assessment session.^23^ Children and their parents each completed the 8-item Pediatric Sleep Disturbance and 8-item Sleep-Related Impairment scales of the Patient-Reported Outcomes Measurement Information System (PROMIS) questionnaire.^35^ The responses provided a subjective assessment of the difficulties the child had in falling and staying asleep (disturbance) and daytime sleepiness and the effects on functioning (impairment). Questionnaire items referred to occurrences over the past week and used 5 frequency response options: never, almost never, sometimes, almost always, or always. The Cronbach α for the sleep disturbance scale was α = 0.87 in children and α = 0.85 in parents. The Cronbach α for the sleep impairment scale was α = 0.91 in children and α = 0.94 in parents.

Children also completed the 27-item KIDSCREEN questionnaire, which assessed HRQOL over the past week, with a score range of 1 (never) to 5 (always) or 1 (not at all) to 5 (extremely), as appropriate.^36^ Children provided answers based on 2 response scales (eg, *Thinking about the last week, have you been in a good mood?* with answer options of never, seldom, quite often, very often, or always; or *Thinking about last week, have you been happy at school?* with answer options of not at all, slightly, moderately, very, or extremely). The KIDSCREEN-27 questionnaire produced a total score (score range: 2.4-5.0) and 5 subscale scores: physical well-being (5 items, with score range: 1.6-5.0; Cronbach α = 0.78), psychological well-being (7 items, with score range: 2.6-5.0; Cronbach α = 0.84), autonomy and parental relations (7 items, with score range: 1.7-5.0; Cronbach α = 0.80), social and peer support (4 items, with score range: 2.0-5.0; Cronbach α = 0.87), and school environment (4 items, with score range: 1.8-5.0; Cronbach α = 0.82). Children were assisted with completing the questionnaires if required; in practice, assistance was rarely needed.

### Statistical Analysis

All analyses were undertaken in Stata, version 17.0 (StataCorp LLC). Effects of mild sleep deprivation were estimated by mixed-effects regression models, with the child as a random effect. Mean differences and 95% CIs were determined for sleep restriction compared with sleep extension. Standardized mean differences (SMDs) and 95% CIs were also calculated using a pooled SD. Residuals of models were plotted and visually assessed for homoskedasticity and normality. Complete case analyses were undertaken using the full sample (intention to treat) and were restricted to those who met the per-protocol a priori definition of a difference in sleep of at least 30 minutes per night.

Two-sided *P* < .05 indicated statistical significance. Data were analyzed between July 4 and September 1, 2022.

## Results

The Figure illustrates that just 5 children withdrew from participation due to health (n = 3) and COVID-19 restrictions (n = 2), leaving a final sample of 100 children. The children included 52 girls (52%) and 48 boys (48%) and had a mean (SD) age of 10.3 (1.4) years; of these children, 24 (24%) had overweight and 16 (16%) had obese status. Of the parents, 47 (47%) reported having a university degree or higher educational level (Table 1).^30,31,32,37^

**Figure.**
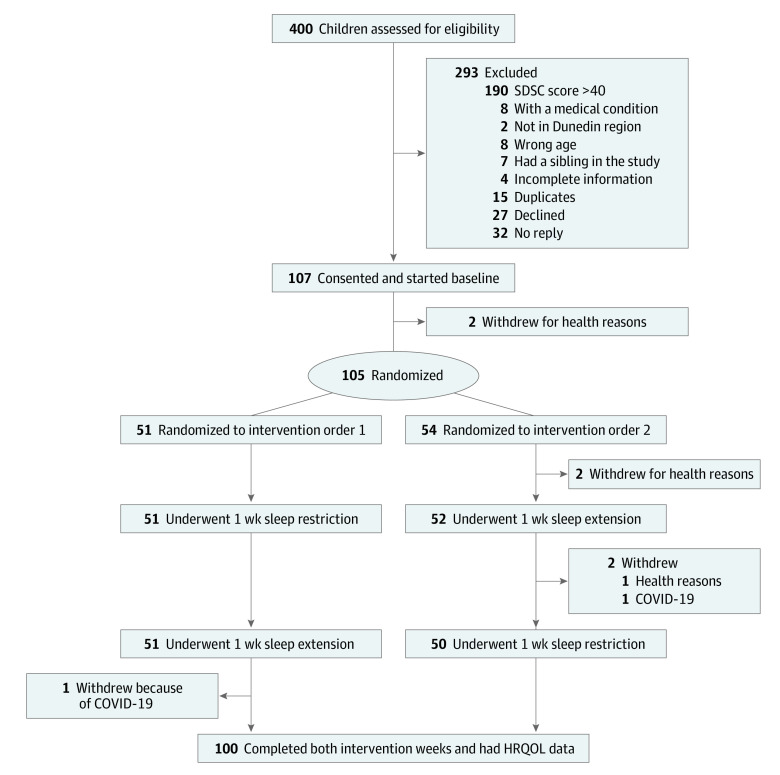
CONSORT Flow Diagram HRQOL indicates health-related quality of life; SDSC, Sleep Disturbance Scale for Children.

**Table 1.** Baseline Demographic Characteristics

Variable	No. (%)
No. of children	100
Sex	
Female	52 (52)
Male	48 (48)
Weight status^a^	
Underweight	3 (3)
Healthy weight	57 (57)
Overweight	24 (24)
Obese	16 (16)
Race and ethnicity^b^	
Asian	4 (4)
Māori	15 (15)
New Zealand European or other^b^	78 (78)
Pacific	3 (3)
Area-level deprivation^c^	
High: NZDep 8-10	20 (20)
Medium: NZDep 4-7	41 (41)
Low: NZDep 1-3	39 (39)
Siblings in study	
Without siblings	86 (86)
With siblings	14 (14)
Maternal educational level^d^	
≤Secondary schooling	26 (26)
Postsecondary education	26 (26)
≥University degree	47 (47)
Missing data	4 (4)
Age, mean (SD), y	10.3 (1.4)
Sleep onset at night, mean (SD), h:min^e^	21:26 (0:40)
Sleep offset in morning, mean (SD), h:min^e^	6:50 (0:35)
Total sleep time, mean (SD), h:min^e^	8:59 (0:45)
WASO, mean (SD), min^e^	23.5 (23.6)
Sleep efficiency, mean (SD), %^e^	95.8 (4.3)
No. of awakenings, mean (SD)^e^	0.71 (0.61)
Children’s Sleep Hygiene Scale, mean (SD)^f^	
Physiological subscale score	5.0 (0.6)
Cognitive subscale score	4.6 (0.8)
Emotional subscale score	5.4 (0.7)
Environmental subscale score	5.6 (0.4)
Bedtime routine subscale score	4.5 (1.1)
Sleep stability subscale score	5.0 (0.4)
Total sleep score	5.0 (0.4)

Abbreviations: NZDep, 2018 New Zealand Index of Deprivation; WASO, wake after sleep onset.

^a^ Categories were based on the World Health Organization body mass index *z* score cutoffs: less than or equal to −2 SDs for underweight, greater than −2 SDs to less than 1 SD for healthy weight, greater than 1 SD to less than or equal to 2 SDs for overweight, and greater than 2 SDs for obese.^32^

^b^ Responses to the New Zealand Census of Population and Dwellings questions allowed prioritization into these specific racial and ethnic categories.^37^ In New Zealand, the “other” category include any other ethnicities.

^c^ Calculated with the 2018 New Zealand Index of Deprivation, which reflected the extent of material and social deprivation in an area and was used to construct deciles from 1 (least deprived) to 10 (most deprived).^30^

^d^ In New Zealand, maternal education is used as the index, and all participating parents or guardians identified as mothers. Secondary schooling in New Zealand covers year 9 through year 13. Postsecondary education refers to all tertiary-level qualifications that are not university based.

^e^ Based on 24-hour actigraphy over 7 days at baseline (N = 100).

^f^ The scale ranged from 1 (never) to 6 (always) or 1 (always) to 6 (never), as appropriate.^31^

Baseline sleep characteristics (from actigraphy) showed that children received a mean (SD) total sleep time of 8 hours and 59 minutes (45 minutes) per night and a high level of sleep efficiency (mean [SD], 95.8% [4.3%]), waking less than once a night on average (mean [SD], 0.71 [0.61] awakenings) (Table 1). Baseline levels of sleep hygiene were good, with mean scores for all subscales of the Children’s Sleep Hygiene Scale being greater than 4 (with 6 as the possible maximum), indicating that the children regularly followed sleep hygiene practices.

Table 2 shows the differences in child sleep between the 2 intervention weeks (with data from the PROMIS questionnaire and actigraphy). With restricted opportunities to sleep, both children (mean difference, −0.6; 95% CI, −0.8 to −0.5) and parents (mean difference, −0.7; 95% CI, −0.8 to −0.5) reported relative reductions in sleep disturbances, suggesting that sleep was more consolidated when sleep opportunities were restricted rather than extended. However, both children (mean difference, 0.4; 95% CI, 0.3-0.6) and parents (mean difference, 0.8; 95% CI, 0.6-0.9) also reported that the child felt more impaired during the day under the sleep restriction condition. Findings were broadly similar when limited to those children who met the a priori difference in sleep across the 2 intervention conditions. The SMDs indicated that the effects on sleep impairment and disturbance were moderate (eg, child-reported sleep impairment: SMD, 0.6; 95% CI, 0.4-0.8) to large (eg, parent-reported sleep disturbance: SMD, –1.1; 95% CI, –1.3 to –0.9).

**Table 2.** Effect of Mild Sleep Deprivation on Subjective Sleep Disturbance and Impairment and Objective Sleep Measures (N = 100)

	No. of participants	Mean (SD)		Mean difference (95% CI)	Standardized mean difference (95% CI)^a^
		Sleep extension	Sleep restriction		
**Full sample**					
PROMIS questionnaire^b^					
Child reported					
Sleep disturbance scale	100	2.3 (0.8)	1.7 (0.7)	−0.6 (−0.8 to −0.5)	−0.8 (−1.0 to −0.6)
Sleep impairment scale	100	1.4 (0.5)	1.8 (0.8)	0.4 (0.3 to 0.6)	0.6 (0.4 to 0.8)
Parent reported					
Sleep disturbance scale	96	2.0 (0.6)	1.3 (0.4)	−0.7 (−0.8 to −0.5)	−1.1 (−1.3 to −0.9)
Sleep impairment scale	99	1.3 (0.4)	2.1 (0.7)	0.8 (0.6 to 0.9)	1.0 (0.8 to 1.1)
Actigraphy^c^					
Sleep onset at night, h:min	96	20:54 (0:45)	21:58 (0:36)	64 (58 to 70)	1.2 (1.1 to 1.4)
Sleep offset in morning, h:min	96	6:37 (0:33)	6:55 (0:32)	18 (13 to 24)	0.6 (0.4 to 0.7)
Total sleep time, min^d^	96	556 (40)	517 (41)	−39 (−46 to −32)	−0.9 (−1.0 to −0.7)
Sleep variability, %^e^	96	10.0 (4.4)	8.9 (5.5)	−1.1 (−2.4 to 0.2)	−0.2 (−0.5 to 0.1)
Weekday-to-weekend differences, min	88	−8.3 (60.5)	−13.9 (53.7)	−5.5 (−22.3 to 11.3)	−0.1 (−0.4 to 0.2)
WASO, min	96	26.2 (23.7)	17.5 (18.0)	−8.7 (−12.9 to −4.4)	−0.4 (−0.6 to −0.2)
Sleep efficiency, %^f^	96	95.6 (4.0)	96.7 (3.3)	1.2 (0.4 to 1.9)	0.3 (0.1 to 0.5)
No. of awakenings	96	0.7 (0.6)	0.5 (0.5)	−0.2 (−0.3 to −0.1)	−0.3 (−0.6 to −0.1)
**Per-protocol sample**					
PROMIS questionnaire^b^					
Child reported					
Sleep disturbance scale	59	2.4 (0.7)	1.8 (0.7)	−0.6 (−0.8 to −0.4)	−0.8 (−1.0 to −0.5)
Sleep impairment scale	59	1.4 (0.5)	1.9 (0.8)	0.4 (0.2 to 0.7)	0.6 (0.3 to 0.9)
Parent reported					
Sleep disturbance scale	56	2.0 (0.7)	1.3 (0.3)	−0.7 (−0.9 to −0.6)	−1.2 (−1.4 to −0.9)
Sleep impairment scale	59	1.3 (0.4)	2.1 (0.7)	0.8 (0.6 to 1.0)	1.0 (0.8 to 1.13)
Actigraphy^c^					
Sleep onset at night, h:min	59	20:53 (0:45)	22:05 (0:36)	71 (64 to 78)	1.3 (1.2 to 1.4)
Sleep offset in morning, h:min	59	6:40 (0:32)	6:49 (0:32)	10 (4 to 16)	0.3 (0.1 to 0.5)
Total sleep time, min^d^	59	563 (40)	502 (36)	−61 (−68 to −54)	−1.3 (−1.4 to −1.1)
Sleep variability, %^e^	59	9.0 (3.6)	9.3 (6.1)	0.3 (−1.4 to 2.0)	0.0 (−0.4 to 0.3)
Weekday-to-weekend differences, min	54	−9.2 (52.8)	−14.2 (52.2)	−5.0 (−24.4 to 14.4)	−0.1 (−0.4 to 0.3)
WASO, min	59	23.7 (22.4)	19.3 (17.1)	−4.5 (−9.7 to 0.8)	−0.2 (−0.5 to 0.0)
Sleep efficiency, %^f^	59	96.0 (3.8)	96.3 (3.2)	0.3 (−0.6 to 1.2)	0.1 (−0.2 to 0.3)
No. of awakenings	59	0.7 (0.5)	0.6 (0.5)	−0.1 (−0.2 to 0.1)	−0.2 (−0.4 to 0.1)

Abbreviations: PROMIS, Patient-Reported Outcomes Measurement Information System; WASO, wake after sleep onset.

^a^ Calculated using a pooled SD.

^b^ Scale ranged from 1 (never) to 5 (always).^35^

^c^ Based on 24-hour actigraphy over 7 days during each intervention week, average-weighted for weekends and weekdays.

^d^ Difference between sleep onset and sleep offset minus any time awake after sleep onset.

^e^ Coefficient of variation in total sleep time.

^f^ Calculated as total sleep time divided by sleep period time (difference between sleep onset and sleep offset) as a percentage.

Accelerometry data indicated that children went to sleep an average of 64 (95% CI, 58-70) minutes later each night over the sleep restriction week compared with the sleep extension week, with a smaller difference (as anticipated) in sleep offset (mean difference, 18; 95% CI, 13-24 minutes). These differences in sleep timing meant that children received 39 (95% CI, 32-46) minutes less of total sleep time each night during the sleep restriction week. These differences were magnified in the per-protocol sample, which had a nightly difference in total sleep time of 71 (95% CI, 64-78) minutes. As observed from the child and parent reports, more consolidated sleep was apparent, with small reductions in the number of awakenings (mean difference, −0.2; 95% CI, −0.3 to −0.1) and wake after sleep onset (mean difference, −8.7 minutes; 95% CI, −12.9 to −4.4 minutes) as well as a small improvement in sleep efficiency (mean difference, 1.2%; 95% CI, 0.4%-1.9%) in the full sample (Table 2). By contrast, sleep variability (variation in total sleep time or difference in total sleep time between weekdays and weekends) did not differ significantly. Significant differences in sleep consolidation were not apparent in the per-protocol sample for any measure.

Table 3 presents the mean differences for HRQOL in the sleep restriction week compared with the sleep extension week. Children reported significantly lower scores for physical well-being (SMD, −0.28; 95% CI, −0.49 to −0.08) and ability to cope well in the school environment (SMD, −0.26; 95% CI, −0.42 to −0.09), leading to total HRQOL scores that were significantly lower when tired (SMD, −0.21; 95% CI, −0.34 to −0.08). Reductions in psychological well-being (mean difference, −0.09; 95% CI, −0.19 to 0.02), social and peer support (mean difference, −0.13; 95% CI, −0.26 to 0.01), and autonomy and parental relations (mean difference, −0.08; 95% CI, −0.19 to 0.03) were observed but were not statistically significant. Differences in HRQOL were generally magnified in the per-protocol sample, with the reduction in social and peer support (SMD, −0.24; 95% CI, −0.47 to −0.01) also being statistically significant. The SMDs indicated that these effects on HRQOL were small (eg, total well-being: SMD, –0.21; 95% CI, –0.34 to –0.08).

**Table 3.** Effect of Restricting and Extending Sleep on Health-Related Quality of Life (N = 100)

	No. of participants	Mean (SD)		Mean difference (95% CI)^a^	Standardized mean difference (95% CI)^b^
		Sleep extension	Sleep restriction		
**Full sample**					
KIDSCREEN-27 questionnaire^c^					
Physical well-being subscale score	99	3.8 (0.7)	3.7 (0.7)	−0.20 (−0.34 to −0.05)	−0.28 (−0.49 to −0.08)
Psychological well-being subscale score	98	4.3 (0.5)	4.2 (0.6)	−0.09 (−0.19 to 0.02)	−0.15 (−0.32 to 0.03)
Autonomy and parental relations subscale score	97	3.9 (0.8)	3.8 (0.8)	−0.08 (−0.19 to 0.03)	−0.10 (−0.25 to 0.04)
Social and peer support subscale score	100	4.3 (0.8)	4.2 (0.8)	−0.13 (−0.26 to 0.01)	−0.15 (−0.32 to 0.01)
School environment subscale score	97	4.2 (0.7)	4.0 (0.8)	−0.19 (−0.31 to −0.07)	−0.26 (−0.42 to −0.09)
Total score	100	4.1 (0.6)	4.0 (0.6)	−0.12 (−0.20 to −0.04)	−0.21 (−0.34 to −0.08)
**Per-protocol sample**					
KIDSCREEN-27 questionnaire^c^					
Physical well-being subscale score	58	3.8 (0.7)	3.6 (0.7)	−0.20 (−0.38 to −0.01)	−0.29 (−0.57 to −0.01)
Psychological well-being subscale score	57	4.3 (0.5)	4.2 (0.7)	−0.11 (−0.25 to 0.03)	−0.18 (−0.42 to 0.06)
Autonomy and parental relations subscale score	57	3.9 (0.7)	3.8 (0.9)	−0.12 (−0.26 to 0.02)	−0.14 (−0.32 to 0.03)
Social and peer support subscale score	59	4.4 (0.7)	4.2 (0.8)	−0.19 (−0.37 to −0.01)	−0.24 (−0.47 to −0.01)
School environment subscale score	58	4.2 (0.7)	3.9 (0.9)	−0.21 (−0.38 to −0.05)	−0.27 (−0.48 to −0.06)
Total score	59	4.1 (0.6)	3.9 (0.6)	−0.15 (−0.26 to −0.04)	−0.25 (−0.43 to −0.06)

^a^ Calculated as sleep restriction compared with sleep extension.

^b^ Effect sizes were standardized using a pooled SD.

^c^ Scale ranged from 1 (never) to 5 (always) or 1 (not at all) to 5 (extremely), as appropriate.^36^ Subscale scores ranged from 1.6 to 5.0 for physical well-being, 2.6 to 5.0 for psychological well-being, 1.7 to 5.0 for autonomy and parental relations, 2.0 to 5.0 for social and peer support, 1.8 to 5.0 for school environment, and 2.4 to 5.0 for total score.

## Discussion

Results of this secondary analysis of the DREAM trial demonstrated that even relatively small reductions in nightly sleep duration can have a considerable effect on HRQOL in children. These children received 39 minutes less sleep per night between sleep conditions over only 1 week. This loss of sleep resulted in significant reductions in the children’s physical well-being, overall well-being, and ability to cope well in a school environment. For those who achieved the a priori difference in sleep of at least 30 minutes per night, additional reductions in well-being associated with less social and peer support were also observed. While these differences may generally be considered as small but not trivial,^38^ the reductions in multiple aspects of HRQOL were observed after only 1 week of less sleep. As such, we believe these findings are clinically and statistically significant and require confirmation over the longer term.

It is difficult to compare the findings with those reported in the literature because most previous experimental studies^12,13^ appeared to involve children with clinical sleep issues, where greater benefits might be expected. In young children with obstructive sleep apnea, significant improvements in HRQOL were observed, principally in school and physical function domains, after adenotonsillectomy.^9^ In children with milder sleep issues (several parent-reported moderate sleep problems at school entry), a brief clinician-delivered intervention showed short-term (3-month) improvements in psychosocial HRQOL even though the proportion of children with sleep problems had not decreased. However, benefits to HRQOL were not maintained at follow-up (12 months), and any corresponding changes in sleep duration were not reported.^39^ Other research that manipulated sleep in children focused more on emotional or cognitive outcomes rather than on HRQOL, reporting consistent adverse effects on mood and smaller effects on emotion and some cognitive test scores.^4,12,13^ We believe the findings of this trial add considerable value to the existing cross-sectional literature that shows the association of sleep duration with HRQOL in children^17,18,19^ because causality can now be inferred.

Previous analyses of the DREAM trial provided some insight into why these relatively modest changes in sleep might affect HRQOL, although it is difficult to disentangle which behavioral changes might influence HRQOL the most as they tend to be interrelated in children.^40^ We found that when children slept less, they ate substantially more calories, particularly in the evenings, all of which came from noncore foods (generally those with poor nutritional quality) rather than from core foods, such as fruit and vegetables,^41^ which are associated with higher HRQOL.^42^ Children replaced this loss of sleep mostly with sedentary time and, to a lesser degree, light activity.^43^ Given that these activity patterns were measured using accelerometry, we do not know which specific behaviors might have changed, although anecdotally, parents reported more screen time during the sleep restriction week. In general, greater HRQOL has been associated with higher amounts of activity and lower amounts of sedentary time in children.^44,45^

### Strengths and Limitations

This study has several strengths. These strengths primarily centered on the randomized crossover trial design, wherein each child participant was able to act as their own control, with the sleep extension week providing the conditions for more sleep opportunity compared with the sleep restriction week that provided less sleep opportunity. We determined the difference in sleep on the basis of actigraphy rather than subjective measures of sleep duration, which should provide greater accuracy.^20^ However, it was interesting to observe greater sleep efficiency when sleep was restricted, suggesting more consolidation of sleep, which was supported by children and parent reports of less sleep disturbance in the PROMIS questionnaire. This finding likely reflects an increased homeostatic drive to sleep as a consequence of sleep restriction.^46^ It is important to note that we specifically tested sleep restriction vs sleep extension conditions to ensure the best opportunity to create a difference in true sleep, as the DREAM trial was a mechanistic study that aimed to determine the effect of mild sleep deprivation on eating and activity behaviors associated with obesity in children.^23,24^ We purposely chose to restrict sleep by a relatively small amount in an effort to mimic clinical levels of mild sleep deprivation,^22^ which we believed had greater applicability for public health than more severe sleep deficits often used in other sleep manipulation trials.^47,48^

This study also has limitations. It focused on a secondary outcome of interest, for which we did not undertake specific power calculations. Although data collection had to be concluded slightly earlier than anticipated due to COVID-19 restrictions, the total dropout rate was low at 5% compared with the projected rate of at least 20%,^23^ ensuring a robust sample size and reduced risk of attrition bias. The sample was not diverse, which may limit extrapolation to other groups. As the trial was a mechanistic study, we cannot comment on the effect of sleep loss over the long term and its implications for HRQOL in children because a consistently linear association between less sleep and worse HRQOL cannot be assumed in the absence of such evidence. Furthermore, we had a single measure of HRQOL from the children; confirmatory data from different measures or parents would have been an advantage.

## Conclusions

In this secondary analysis of the DREAM randomized crossover trial of sleep manipulation, we showed that after only 1 week of receiving 39 minutes less sleep per night between sleep conditions, children reported significantly lower HRQOL in terms of their physical and overall well-being and ability to cope well at school. These findings highlight that ensuring children receive sufficient good-quality sleep is an important child health issue.

## Notes

Supplement 1.
Protocol

Supplement 2.
Data Sharing Statement
