# Supplementary material for: Effect of Sleep Changes on Health-Related Quality of Life in Healthy Children: A Secondary Analysis of the DREAM Crossover Trial
Source: JAMA Netw Open. 2023 Mar 15;6(3):e233005. doi: 10.1001/jamanetworkopen.2023.3005 (PMC10018327; doi:10.1001/jamanetworkopen.2023.3005)
Supplement: Supplement 1. — Protocol [file jamanetwopen-e233005-s001.pdf]

Protocol

Study title: The Daily Rest, Eating, & Activity Monitoring Study

Study investigators:

Principle Investigator: Professor Rachael Taylor
Department of Medicine, University of Otago
rachael.taylor@otago.ac.nz

Co-investigators: Professor Barbara Galland
Department: Women's and Children's Health, University of Otago
barbara.galland@otago.ac.nz

Dr Jill Haszard, Biostatistician
Biostatistics Unit, University of Otago
jill.haszard@otago.ac.nz

Dr Kim Meredith-Jones
Department of Medicine, University of Otago
kim.meredith-jones@otago.ac.nz

Professor Dean Beebe
Department of Neuropsychology, Cincinnati Children's Hospital Medical Center, USA
beebd0@cchmc.org

Professor Dawn Elder
Department of Pediatrics, University of Otago Wellington
dawn.elder@otago.ac.nz

Study co-ordinators: Dr Aimee Ward
Department of Medicine, University of Otago
aimee.ward@otago.ac.nz

Ms Deborah McIntosh
Department of Medicine, University of Otago
deborah.mcintosh@otago.ac.nz

PhD candidates: Ms Silke Morrison
Department of Medicine, University of Otago
silke.morrison@postgrad.otago.ac.nz

Ms Rosie Jackson
Department of Medicine, University of Otago
rosie.jackson@postgrad.otago.ac.nz

1. Lay summary

One in three NZ children are overweight or obese and not getting enough sleep may be part of the problem. However, while we know that insufficient sleep is a strong risk factor for obesity, we don't know why. Research suggests that it changes what we eat, but intervention studies are required to determine whether this is true. This study will produce mild sleep deprivation in 8-12 year old children over one week and measure what effect this has on their appetite, what/when and how they eat, and their participation in physical activity and sedentary behaviour (e.g. screen time).

2. Background

If a child has a regular habit of forgetting to take important items to school each day, we might wonder if he/she is getting enough sleep –but if the same child, after a full breakfast, regularly devours a hot pie on the way to school, would we consider the same? Experiments in adults suggest we should. Acute (1 night) or chronic (several days) sleep restriction can increase calorie intake[1] and alter food purchasing in favour of higher-caloric foods.[2] There is also some evidence that endocrine drivers of appetite regulation work in favour of increased food consumption[3] and reward[4] suggesting both homeostatic and hedonistic mechanisms at play. These types of experimental studies provide insight into potential pathways mediating the well-established relationship between short sleep and high risk of obesity in adults.[5]. However, while this relationship is even stronger in children than in adults [6,7], a very limited body of research has examined how sleep deprivation influences food and eating behavior in children. The two existing studies show that 1-5 nights of restricted sleep increases energy intake by 7-21%[8,9], but their small size (n=10 and 37) limits any examination of how diet changes when children are sleep-deprived. Because obese children present with fewer comorbidities than adults, this suggests that children may be better models to further our understanding of how sleep loss may impact food intake and eating behaviour.

3. Design and aims

This experimental study aims to determine how mild sleep deprivation might influence eating behaviour and activity patterns in children. As it is not known exactly how much sleep each individual child needs,[10] all children will undergo two experimental sleep conditions. In the restriction week, the child will go to bed 1 hour later than they normally do, while maintaining usual wake up time (therefore hopefully sleeping up to one hour less than normal). In the extension week, the child will go to bed 1 hour earlier than they normally do, while maintaining usual wake up time (therefore hopefully sleeping up to one hour more than normal). All analyses compare the difference between these two experimental weeks. Because not all children will change sleep by exactly one hour in each condition, we expect that the difference between the two conditions should produce a reduction in "usual" sleep time of 1-2 hours per night for each child. This level of mild sleep deprivation is thought to be common in children.[11]

The **primary aim** is to determine whether mild sleep deprivation:

- Increases eating in the absence of hunger (measured objectively).

Secondary aims will determine whether mild sleep deprivation:

1) Increases the amount and types of foods eaten, especially before bed.
2) Alters the *context* of eating (where, when, who with).

3) Changes other indices of eating behaviour (e.g. emotional over-eating).
4) Changes children's preference for different food types.
5) Increases time spent sedentary or in physical activity.

4. Participants

Children aged 8 to 12 years will be recruited through community networks, approaches to interested schools, and Facebook (https://www.facebook.com/Otago.Sleep.Research/). We aim to recruit a sample of children that is broadly representative of New Zealand children to increase the generalisability of findings (e.g. 50% female, 30% overweight/obese, 60% European, 21% Māori, 11% Pacific, 11% Asian). Children with a wide range of 'normal' sleep patterns will be recruited. Those who are interested will be asked to contact us via e-mail or phone. Potential participants and parents of potential participants will then be directed to an online screening questionnaire to provide demographic information and (child date of birth, date of survey, household address to calculate household deprivation, ethnicity, family structure), and child sleep and health information, to assess eligibility as follows:

5. Eligibility criteria

Inclusion criteria:
• Between 8 and 12 years
• Reported time in bed of 8-11 hours per night
• Normal sleep patterns

Exclusion criteria:
• Medical condition or medication that affects sleep or eating behaviour
• Presence of sleep disturbances.

Only children with reported time in bed (time between lights out and waking in the morning) of 8-11 hours a night will be eligible. This will ensure that any sleep extension or restriction does not place them in the "not recommended" category for sleep duration according to international guidelines (< 7 or > 12 hrs).[12] The well-validated Sleep Disturbance Scale for children[13] will identify children with potential sleep problems such as sleep-walking, restless legs, sleep disordered breathing, problems with initiating and maintaining sleep, and excessive sleepiness. Children identified as having a sleep disorder, or those with any chronic medical condition or physical disability that impedes their ability to participate in physical activity, will be excluded.

6. Intervention

Following a baseline week to establish "usual" sleep, eligible participants undergo sleep restriction or sleep extension conditions using a randomised, counterbalanced, cross-over design. Measurements occur at various time points as illustrated by the figure:

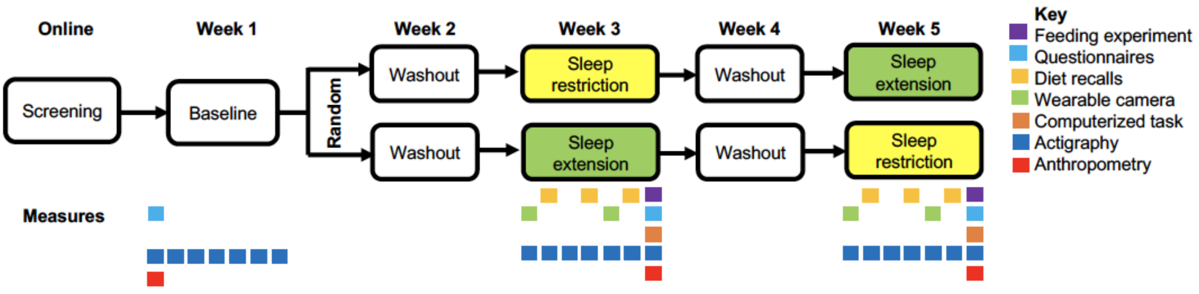

Baseline: Participants will attend a 30-minute baseline appointment at home with their parent(s). After informed consent has been obtained, height and weight will be measured following standard procedures. An Actigraph (wGT3X-BT) accelerometer will be attached to the waist and worn 24-hours a day for one week to measure sleep, physical activity and sedentary behaviour. Parents will complete a baseline questionnaire which assesses demographics, food preferences and allergies (to avoid any issues with the feeding experiment), and sleep hygiene (to enable personalisation of intervention weeks. Participants are provided with a pre-paid courier bag to enable return of the accelerometer at the end of the week.

Weeks 2 and 4: **Nothing for participants**. Researchers determine usual sleep and wake times from the accelerometry data and accompanying sleep diary (parent simply indicates 'lights off' and 'lights on' time each day). Researchers randomise participants to begin with sleep restriction or sleep extension.

Weeks 3 and 5: Researchers discuss the new bedtime with the parent(s) and child on day 1. Those randomised to **sleep extension** will be instructed to go to bed one hour earlier than their usual bed time (determined in week 1) each night for 7 nights, while maintaining the usual wake up time. Those randomised to **sleep restriction** will be instructed to go to bed one hour later than their usual bed time (determined in week 1) each night for 7 nights, while maintaining the usual wake up time. Naps are not allowed. Changing bed time while maintaining wake time reflects the real world where wake times remain relatively constant in children. We will work with parents and children to determine how best to ensure adherence for each family using a variety of strategies as outlined below and utilised by co-investigator Beebe in his studies.[14] Daily personalised text reminders to child and parent will also encourage adherence.

**Behavioral Strategy**	**Description**
**Securing motivation**	Prior to the manipulation, staff will discuss study challenges, giving the family the opportunity to opt out and asking if they want to move forward. Once the child verbalizes the desire to move forward, staff will align with the child and parent around the common mission of success with the protocol.
**Goal setting**	Children will be instructed to increase or decrease their time in bed by 1 hour relative to their baseline sleep duration. Specific bedtime and wake time goals will be set to achieve this.
**Preplanning**	Staff will preplan with the child/parent dyad how to best adhere to the set sleep schedule and duration given the child's unique schedule, demands, and circumstances. This involves reviewing typical schedules and trying to identify sources of inconsistency in those schedules that could affect adherence.
**Problem solving**	Barriers to achieving adherence will be identified with children and parents. Staff will work with families to help identify strategies to overcome barriers and ways in which they could prioritize and reorganize activities to allow for an earlier/later bedtime.

**Positive routine**	Healthy routines leading up to bedtime will be encouraged, including reducing screen time and completing typical before bed activities (eg, showering, brushing teeth) before the set bedtime.
**Sleep hygiene**	Children will be instructed to limit caffeine during the day to one coffee, one energy drink, or two caffeinated sodas with encouragement to avoid caffeine altogether. They will also be instructed to eliminate screen time at bedtime and to turn off cell phones, set them to “airplane mode,” or keep them outside of their bedrooms.
**Self-monitoring**	Children complete daily sleep diaries. They will be informed that the diaries will be reviewed with them and their parent alongside actigraphy data during office visits.
**Positive reinforcement**	When conducting sleep reviews, researchers will positively reinforce children for adhering to prior sleep instructions. Further, parents will be encouraged to support and positively reinforce their child for adherence to the set sleep schedule.

7. Outcome assessments

All outcome measurements will be undertaken by staff blinded to sleep condition where possible. Some measurements occur daily (actigraphy), on several days (diet recalls, cameras, anthropometry) or just at the end of each experimental week (hunger experiment, food cravings, questionnaires).

<u>Primary outcome:</u>

Eating in the absence of hunger

At the end of the assessment session on day 8 of weeks 3 and 5, children are offered a buffet meal and eat ad-libitum until they are full (first course). Participants will be asked to rate their feeling of fullness using the Teddy the Bear scale.[15] After 15 minutes, children are provided with an opportunity to eat highly palatable snacks (e.g. lollies, chips) for 10 minutes without adult supervision (second course). Energy intake from both courses is calculated following weighing of each individual food or beverage item prior to and after consumption. The energy consumed in the second course indicates eating in the absence of hunger.[16]

<u>Secondary outcomes:</u>

Dietary intake (secondary aim 1)

Parental/child dyads will be interviewed about their child’s dietary intake on days 3 and 8. Recalls assess the previous day’s intake i.e. this will assess intake on days 2 and 7). We will use the multiple 24-hour recall method (as used in our National Nutrition Surveys) and interviews will be in person (30 minutes) in the child’s home (day 3) or the University (day 8). This protocol collects detailed information on timing of eating, description of food (including brand names where possible), and amounts consumed. Recall data will be entered into FoodWorks to calculate the energy, macronutrients and micronutrients contained in the recalled diet using FOODfiles, the food composition tables for New Zealand. Collecting two 24-hour recalls will enable us to calculate ‘usual intake’ using the Multiple Source Method for estimating usual dietary intake of individuals.[17]

The context of eating (secondary aim 2)
A small auto-camera (automatically takes a picture every 2 seconds) will be worn on days 2 and 7 during all waking hours on the days prior to collecting the diet recall data. In this way, the camera images assess the same day as the recalls do (as diet recalls assess previous days intake). The images will be viewed by research staff parents contacted for clarity regarding any food intake that appears in the camera images but not the diet recall, where feasible. The images will provide information regarding the environmental context of eating as it occurs in the free-living environment. Extensive guidelines and frameworks have been developed to ensure privacy of participants and third parties.[18] Please see section 9 for a comprehensive description of relevant ethical issues.

Eating behaviour (secondary aim 3)
Parents complete the Child Eating Behaviour Questionnaire (CEBQ),[19] which provides 8 subscales including satiety responsiveness (5 items, how well they eat to appetite), food responsiveness (5 items, eating for reasons other than hunger), emotional over-eating (4 items, eating because of emotions), and fussiness (6 items, α 0.74-0.91). As this questionnaire typically assesses "usual" behaviours, response answers will be adapted to reflect past week.

Preferences for different foods (secondary aim 4)
During the assessment session on day 8, children are shown pictures of a variety of foods differing in nutritional quality and asked to rate how much they would like to eat each food right now on a sliding scale between "not at all" to "heaps".

Time spent in physical activity, sedentary behaviour and sleep (secondary aim 5)
An Actigraph (wGT3X-BT) accelerometer will be attached to the waist and worn 24-hours a day for one week to measure sleep, physical activity and sedentary behaviour at baseline, and during each intervention week. Actigraphs will be initialized using 15-second epochs with ActiLife Software (Version 9.0.0). Parents will complete sleep diaries that indicate "lights off" and "lights on". Sleep onset (child goes to sleep) and offset (child wakes), and total sleep time (TST, time between sleep onset and offset, minus time awake in the night) will be calculated using a validated automated count-scale algorithm written in MatLab® (MathWorks, Natick, MA,USA)[20]. Once sleep has effectively been removed from the data, time spent in physical activity and sedentary time will be calculated using appropriate cutoffs.[21]
Other measures
Several measures of potential adverse outcomes are obtained: 1) children complete the Kidscreen questionnaire which assesses mood and quality of life over the past week,[22] 2) children complete the PROMIS questionnaire[23] which assesses sleep disturbances (difficulties falling and staying asleep) and sleep impairment (impact on daytime functioning) over the past week, and 3) parents a proxy version of the PROMIS questionnaire (to provide different perspective of the outcomes).

8. Statistical considerations and data analysis
Sample size: Our primary outcome is to determine the difference in eating in the absence of hunger when mildly sleep deprived. For the objective experiment, based on a standard deviation of 870kJ and a within-person correlation of 0.7,[24] a sample size of 59 would be

required to detect a difference of 250 kJ in energy intake between the two different sleep conditions (80% power, p<0.05). 250kJ is equivalent to approximately 1.5 plain biscuits or 6 potato crisps. These numbers will also allow us to detect important differences in energy intake (secondary outcome) from the diet recalls, over the total day (500kJ difference, n=85) or just at night (200kJ difference, n=50). Recruiting 110 children will allow for a representative sample, with 20%-40% drop-out, incomplete data, or not meeting the required sleep difference of 30 minutes for per protocol analyses.

Data analysis: Mixed effects regression models will be used to determine mean differences between the two experimental conditions (sleep restriction and sleep extension) with participant ID as a random effect which accounts for both within-person and between-person variation. Skewed data will be log-transformed as appropriate. For binary outcomes, generalised estimating equations will be used to determine odds ratios between conditions. 24-hour movement data (actigraphy) will be analysed using fractional multinomial logit models to account for the compositional nature of the data. Intention-to-treat analyses will be undertaken as well as per-protocol analyses, where ‘per protocol’ will be the sample with at least 30 minutes sleep difference between restriction and extension (representing mild sleep deprivation).

9. Ethical considerations

Wearable cameras: Auto-cameras are increasingly being used for health research but such devices can also be somewhat intrusive. Fortunately, there are previous research groups that have recommended ethical frameworks to guide their use.[18] including University of Otago based research.[25]

Associate Professor Louise Signal’s group adopted a framework that consolidated the ethical issues raised in numerous studies and applications for ethical approval, and addressed protocols that uphold key ethical principles. The key issues relate to the legality of recording images in public places; the potential to capture images of illegal activity in which the wearer is either participating or witnessing; third party consent to be photographed; parental consent for recording of images in the home environment; ownership of participant-generated images; data handling and storage; privacy and anonymity of participants and third parties; and participant safety.

• **Taking images in public places:** New Zealand privacy laws state that in New Zealand it is generally lawful to take photographs of people in public places without their consent, so long as they are in a place where there is no expectation of privacy, such as a beach, shopping mall, park or other public place. However, photographs must not be taken if participants are in a place where people would expect reasonable privacy (such as public toilets and changing areas), where the publication would be highly offensive to an objective and reasonable person; when there is potential to stop other people's use and enjoyment of the same place; or there is no legitimate reason for taking the photos (New Zealand Police, 2012 (http://www.police.govt.nz/faq/items/23297). In this instance, participants would be advised by researchers to turn the cameras off or turn them around. Participants will be given a pre-prepared statement about the study. If approached, they would be advised to explain that they are participating in a study being conducted by researchers from the University of Otago; that the project aims to document their environment; and that they are

wearing a camera that automatically takes pictures continually throughout the day. Furthermore, they would be advised to say that they are not intentionally taking photographs of specific people or places. They would also be encouraged to tell interested parties to contact the researchers if they have additional information or have further questions. The consensus from previous projects was that most people were unconcerned with the device's presence after an explanation was given. In cases where concern might be raised, the camera can be simply removed or covered.

• **Protecting the home environment:** Parent/guardians will be briefed on when it will be necessary to remove the camera to protect their own privacy and the privacy of members of the public. This will also be outlined in the written material provided to them.

• **Third party consent:** It is impractical to obtain informed consent from every member of the public within the study location. The Third parties are not the intended subjects of the images. To protect the privacy of those who may be inadvertently captured in the images, all images used in disseminated material will have identifiable people, street names, or other identifying signs blurred following data collection. DSM ITS will provide guidance on this aspect. Thus, we feel that the privacy of those who have not consented to be part of the study will be protected through these actions.

• **Capture of illegal activity:** Automated camera devices also have the potential to capture images of illegal activity that the wearer is either participating in or witnessing. Although the capture of incriminating images has been discussed in the international literature, there is minimal literature regarding the legal obligations of visual researchers in the New Zealand context. Legal advice sought from the Faculty of Law, University of Otago, Dunedin (Assoc. Prof. Margaret Briggs) by the KidCam project in 2013 indicated that in the unlikely event that illicit activity is captured involving others, we would be obligated to pass these on to the Police. If this situation arises, legal advice will be sought. Confidentiality: All images passed on to the research team will be treated as confidential material. Participants will be informed of this on the information sheet and consent form.

• **Privacy**: Participants will be comprehensively briefed at the beginning of the data collection period about situations in public and private in which it would be inappropriate to continue taking photographs and the device needs to be removed/reversed to cover the lens or turned off. Of particular concern to this project is the capturing of screen use. The camera may take photos of sensitive emails and social media posts from third parties. Participants will have first access to the images and delete any they do not want the researchers to see.

• **Ownership and the use of participant generated images in research**: To prevent the images being released into the public domain by the participants, either in print form or via the internet, transfer of ownership is necessary to safeguard the privacy and anonymity of the participants and any other persons that appear in the images. A small number of participant generated images may also be used in material that is published, presented or otherwise disseminated. As a condition of participating in this study, participants will be asked to transfer copyright and ownership of their images as part of the consent process.

10. References

1. Brondel L, Romer MA, Nougues PM, Touyarou P, Davenne D. Acute partial sleep deprivation increases food intake in healthy men. *Am J Clin Nutr.* 2019;91(6):1550-1559.
2. Chapman CD, Nilsson EK, Nilsson VC, et al. Acute sleep deprivation increases food purchasing in men. *Obesity.* 2013;21(12):E555-E560.

3. Speigel K, Tasali E, Penev P, Van Cauter E. Sleep curtailment in healthy young men is associated with decreased leptin levels, elevated ghrelin levels, and increased hunger and appetite. *Ann Intern Med.* 2004;141(11):846-850.
4. St-Onge MP, McReynolds A, Trivedi ZB, Roberts AL, Sy M, Hirsch J. Sleep restriction leads to increased activation of brain regions sensitive to food stimuli. *Am J Clin Nutr.* 2012;95(4):818-824.
5. Ogilvie RP, Patel SR. The epidemiology of sleep and obesity. *Sleep Health.* 2017;3(5):383-388.
6. Carter PJ, Taylor BJ, Williams SM, Taylor RW. A longitudinal analysis of sleep in relation to BMI and body fat in children: the FLAME study. *BMJ.* 2011;342:d2717.
7. Miller MA, Kruisbrink M, Wallace J, Ji C, Cappuccio FP. Sleep duration and incidence of obesity in infants, children, and adolescents: a systematic review and meta-analysis of prospective studies. *Sleep.* 2018;41(4):zsy018. https://doi.org/010.1093/sleep/zsy1018.
8. Hart CN, Carskadon MA, Considine RV, et al. Changes in children’s sleep duration on food intake, weight, and leptin. *Pediatrics.* 2013;132:1-8.
9. Mullins EN, Miller AL, Cherian SS, et al. Acute sleep restriction increases dietary intake in preschool-aged children. *J Sleep Res.* 2017;26:48-54.
10. Blunden S, Galland B. The complexities of defining optimal sleep: empirical and theoretical considerations with a special emphasis on children. *Sleep Med Rev.* 2014;18:371-378.
11. Short MA, Blunden S, Rigney G, et al. Cognition and objectively measured sleep duration in children: A systematic review and meta-analysis. *Sleep Health.* 2018;4(3):292-300.
12. Hirshkowitz M, Whiton K, Albert SM, et al. National Sleep Foundation’s sleep time duration recommendations: methodology and results summary. *Sleep Health.* 2015;1(1):40-43.
13. Bruni O, Ottaviano S, Guidetti V, et al. The Sleep Disturbance Scale for Children (SDSC). Construction and validation of an instrument to evaluate sleep disturbances in childhood and adolescence. *J Sleep Res.* 1996;5:251-261.
14. Van Dyk TR, Zhang N, Catlin PA, et al. Feasibility and emotional impact of experimental extending sleep in short-sleep adolescents. *Sleep.* 2017;40(9):DOI. 10.1093.
15. Bennett C, Blissett J. Measuring hunger and satiety in primary school children. Validation of a new picture rating scale. *Appetite.* 2014;78:40-48.
16. Lansigan RK, Emond JA, Gilbert-Diamond D. Understanding eating in the absence of hunger among young children: a systematic review of existing studies. *Appetite.* 2015;85:36-47.
17. Harttig U, Haubrock J, Knuppel S, Boeing H, EFCOVAL Consortium. The MSM program: web-based statistics package for estimating usual dietary intake using the Multiple Source Method. *Europ J Clin Nutr.* 2011;65(Suppl 1):S87-S91.
18. Kelly P, Marshall SJ, Badland H, et al. An ethical framework for automated, wearable cameras in health behavior research. *Am J Prev Med.* 2013;44(3):314-319.
19. Wardle J, Guthrie CA, Sanderson S, Rapoport L. Development of the Children's Eating Behaviour Questionnaire. *J Child Psychol Psychiatry.* 2001;42:963-970.
20. Smith C, Galland BC, Taylor RW, Meredith-Jones KA. ActiGraph GT3X+ and Actical wrist and hip worn accelerometers for sleep and wake indices in young

children using an automated algorithm: validation with polysomnography. *Frontiers in Psychiatry; Sleep Disorders.* 2019;10:958.

21. Evenson KR, Catellier DJ, Gill K, Ondrak KS, McMurrary RG. Calibration of two objective measures of physical activity for children. *Journal of Sport Sciences.* 2008;26(14):1557-1565.
22. Ravens-Sieberer U, Herdman M, Devine J, et al. The European KIDSCREEN approach to measure quality of life and well-being in children: development, current application, and future advances. *Qual Life Res.* 2014;23(3):791-803.
23. Forrest CB, Meltzer LJ, Marcus CL, et al. Development and validation of the PROMIS Pediatric Sleep Disturbance and Sleep-Related Impairment item banks. *Sleep.* 2018;zsy054:https://doi.org/10.1093/sleep/zsy1054.
24. Hill C, Llewellyn CH, Saxton J, et al. Adiposity and 'eating in the absence of hunger' in children. *Int J Obes.* 2008;32:1499-1505.
25. Barr M, Signal L, Jenkin G, Smith M. Capturing exposures: using automated cameras to document environmental determinants of obesity. *Health Promotion International.* 2015;30(1):56-63.
