# Supplementary material for: Effect of Sleep Changes on Health-Related Quality of Life in Healthy Children: A Secondary Analysis of the DREAM Crossover Trial
Source: JAMA Netw Open. 2023 Mar 15;6(3):e233005. doi: 10.1001/jamanetworkopen.2023.3005 (PMC10018327; doi:10.1001/jamanetworkopen.2023.3005)
Supplement: Supplement 2. — Data Sharing Statement [file jamanetwopen-e233005-s002.pdf]

# Data Sharing Statement

Taylor. Effect of Sleep Changes on Health-Related Quality of Life in Healthy Children. *JAMA Netw Open*. Published March 15, 2023. doi:10.1001/jamanetworkopen.2023.3005

**Data**
**Data available:** Yes
**Data types:** Deidentified participant data, Data dictionary
**How to access data:** Please contact the corresponding author at rachael.taylor@otago.ac.nz
**When available:** beginning date: 01-01-2024, end date: 12-31-2024

**Supporting Documents**
**Document types:** None

**Additional Information**
**Who can access the data:** The data will be made available upon reasonable request to the corresponding author
**Types of analyses:** For meta-analyses
**Mechanisms of data availability:** After approval of a proposal
